# Transitioning from river blindness control to elimination: steps toward stopping treatment

**DOI:** 10.1093/inthealth/ihx049

**Published:** 2018-02-19

**Authors:** Paul T Cantey, Sharon L Roy, Daniel Boakye, Upendo Mwingira, Eric A Ottesen, Adrian D Hopkins, Yao K Sodahlon

**Affiliations:** 1 Department of Neglected Tropical Diseases, World Health Organization, Geneva 1211, Switzerland; 2 Division of Parasitic Diseases and Malaria, U.S. Centers for Disease Control and Prevention, Atlanta, GA 30329, USA; 3 Parasitology Department, Noguchi Memorial Institute for Medical Research, Accra, LG581, Ghana; 4 Neglected Tropical Diseases Control Programme, Ministry of Health, Community Development, Gender, Elderly and Children, Dar es Salaam 11478, Tanzania; 5 National Institute for Medical Research, Dar es Salaam 11101, Tanzania; 6 Task Force for Global Health, Atlanta, GA 30030, USA; 7 Independent Consultant, Gravesend DA1, UK; 8 Mectizan Donation Program, Atlanta, GA 30030, USA

**Keywords:** Elimination, Monitoring and Evaluation, Onchocerciasis

## Abstract

The transition from onchocerciasis control to elimination requires country programmes to rethink their approach to a variety of activities as they move from addressing morbidity to addressing transmission of the parasite. Although the 2016 WHO guidelines provide extensive recommendations, it was beyond the scope of the document to provide guidance on all aspects of the transition. This paper will discuss some of the important issues that programmes are grappling with as they transition to elimination and provide some potential approaches that programmes can use to address them. Although there are some data to support some aspects of the suggested approaches, operational research will be needed to generate data to support these approaches further and to determine how programmes could best tailor them to their own unique epidemiological challenges. Good communication between the national programmes and the broader global programme will facilitate the clear articulation of programmatic challenges and the development of the evidence to support programme decision-making.

## Introduction

River blindness, or onchocerciasis, is caused by the filarial nematode *Onchocerca volvulus*, which is transmitted by the bite of infected blackflies from the *Similium* genus. After ivermectin was made available for the prevention of onchocerciasis-related blindness by the Mectizan Donation Program in 1987, affected countries together with the support of non-governmental partners and the WHO began annual community-wide distribution of the medication to prevent blindness and reduce skin disease. Over the years, a variety of regional programmes have supported the onchocerciasis control effort. These include the Onchocerciasis Control Programme for West Africa (OCP), the African Programme for Onchocerciasis Control (APOC), the Onchocerciasis Elimination Program for the Americas (OEPA) and now the Expanded Special Project for the Elimination of Neglected Tropical Diseases in Africa (ESPEN). The impact of the programmes has been substantial^1–4^ and successful interruption of transmission in the Americas^2,5–8^ and Africa^9–11^ has provided the momentum for the transition from morbidity control to the elimination of parasite transmission. Currently, more than 198 million people live in areas that are endemic for the parasite, and nearly 132 million people received treatment with ivermectin in 2016.^1,12^ Treatment is no longer needed for 1.4 million people and has been stopped; four countries have been verified by WHO as having eliminated transmission.^2,6–8^

## A changing mindset

The 2016 WHO *Guidelines for the stopping of mass drug administration and verification of elimination of human onchocerciasis* define three phases of elimination.^13^ Phase 1 is the phase of active transmission and ivermectin mass drug administration (MDA). During Phase 1 programmes should achieve at least a minimum 80% therapeutic coverage of the eligible population for 12–15 years or longer in order to reach a point where transmission of the parasite can no longer be sustained. Achieving higher coverage will shorten the time to interruption of transmission.^14^ At the end of Phase 1, programmes perform epidemiological and entomological evaluations; if the results meet the criteria described in the 2016 guidelines, MDA may be stopped. Programmes then enter Phase 2, which is the 3–5-year period of post-treatment surveillance (PTS). An entomological evaluation is required at the end of PTS to confirm interruption of transmission. If confirmed, the programme enters Phase 3, a period of post-elimination surveillance (PES), the purpose of which is to detect possible recrudescence or reintroduction of onchocerciasis.

The 2016 WHO guidelines require the establishment of an oversight committee by the country Ministry of Health as part of the elimination process.^13^ This oversight committee needs to be independent of the national onchocerciasis programme in order to provide objective advice to the national programme and the Ministry of Health. Although the composition of the committee varies from country to country, members chosen by the Ministry of Health are usually local experts from outside the ministry and international experts. In order to preserve the committee’s independence, national programme officials attend the meetings and may serve on the secretariat, but typically do not vote on committee decisions. The committee’s role is to advise the ministry on several issues:

- Whether the requirements of the guidelines for stopping MDA for onchocerciasis have been met.
- Whether the programme has met the requirements of the guidelines for stopping PTS.
- Whether it is time to initiate the WHO verification of elimination process.

Many of these committees are assuming a much larger role, assisting national programmes with the creation of national elimination plans, which are needed not only to help change the mindset of the programme, but also to address issues not covered in the guidelines. Regardless of the specific role of the committee, the Ministry of Health maintains control over its programme; it may decline to follow the committee’s advice. However, the process of providing recommendations that are reviewed by the Ministry of Health creates a record that can be referred back to when new evidence is developed that could impact the recommendations.

The transition from control to elimination is a complex process, as programmes must move from identifying communities with symptomatic individuals to identifying communities with infected, but asymptomatic individuals; in order to interrupt transmission and prevent its recrudescence, they must find people with the parasite, not just people with the disease. The rapid epidemiological mapping of onchocerciasis (REMO) followed by rapid epidemiological assessments (REAs) were used for the delineation of treatment areas in Africa and were based on the risk of developing blindness. Hypo-endemic areas (defined as having less than 35–40% microfiladermia) had less than a 1% risk of blindness^15^ and thus were not targeted for treatment, even if they might have contributed to transmission, because they did not contribute significantly to blinding morbidity. However, elimination requires identification and treatment of all geographic areas that contribute to transmission and achievement of therapeutic drug coverage levels that are maintained for many years not only to prevent morbidity but also to suppress transmission below the threshold required to sustain the parasite population.

## Elimination mapping for scale-up

In order for countries to eliminate the transmission of onchocerciasis, country programmes must implement MDA in all areas where the transmission of the parasite occurs. This first requires elimination mapping, which is an evaluation of the current situation of onchocerciasis endemicity in all areas where transmission may occur and where ivermectin MDA has not been implemented. An initial consideration in elimination mapping is defining the onchocerciasis transmission zone (TZ). The entire TZ needs to be mapped and evaluated. Different approaches are being developed by different programmes, some defining the TZ using administrative units (e.g. district) and others using epidemiological units (e.g. focus or river basin). Either approach requires consideration of where to implement MDA (e.g. does the presence of any transmission in a district require treatment of the entire district?), cross-border issues and the population that should be evaluated during a single transmission assessment.^16^ Programmes need to move away from thinking in terms of hyper-, meso- and hypo-endemic when making decisions on where to implement MDA. Instead the focus needs to be on whether transmission is present or absent. Anywhere transmission is present, treatment needs to be implemented and maintained until interrupted.

Although much of Africa was mapped using the REMO/REA strategy, which relied on nodule palpation, the strategy was not designed to detect low level transmission, but rather to detect areas at >1% risk of blindness.^15^ In fact, data show that areas with no nodules found on examination could still have microfiladermia prevalence of greater than 20%.^17^ More recently, this finding was confirmed when areas that were initially mapped as hypo-endemic by nodule palpation in Gabon were now found to be meso- or hyperendemic when evaluated using skin snips;^18^ the baseline REMO/REA data cannot be used to exclude transmission, both because it was not designed to detect low transmission and because the situation may have changed. Skin snip surveys would allow more precise determination of endemicity of onchocerciasis than REMO/REA, but there are concerns about the sensitivity of the test, particularly in low prevalence settings,^19,20^ and asymptomatic individuals appear to be less inclined to accept skin snips. The most promising diagnostic option is the Ov-16 serological test, which comes in ELISA and rapid diagnostic test (RDT) formats. The RDT would be the easiest to use programmatically, although it may not have adequate sensitivity to allow its effective use in low prevalence settings;^21^ fortunately, work is already underway to evaluate the RDT sensitivity so that sample sizes or thresholds can be adjusted to compensate for the test sensitivity. More information on diagnostic tests for onchocerciasis can be found in this supplement.^22^ In areas where lymphatic filariasis (LF) is co-endemic and where MDA for LF is occurring, elimination mapping could be performed in a manner integrated to or coordinated with the first LF transmission assessment.

## Monitoring and evaluation

The 2016 guidelines specify that routine monitoring and evaluation (M&E) of parasitological, entomological and serological markers should occur at least every 4–5 years according to local regional guidelines. As Ov-16 serology is a component of the data now required to make the decision to stop MDA at the end of Phase 1, programmes will need to transition from using skin snips to using Ov-16 not only for mapping in low TZ, but also for the transmission assessments during routine programme M&E during this phase. Programmes may initially want to obtain skin snips from some or all of the people who are tested by Ov-16 serology in order to understand how the seroprevalence in children changes over time during the course of the programme in comparison with skin snips. As country programmes gain more experience with Ov-16, it will be important to move away from parasitological indicators (e.g. skin snip) and substitute Ov-16 serology in children as a parasitological indicator over the next 1–2 years. Harmonization of the available Ov-16 ELISAs and standardized comparisons with the RDT are underway. Currently, either format can be used for M&E, and the RDT format can provide rapid results for programmes. However, negative results by RDT should be interpreted with caution or confirmed with ELISA until the comparisons between ELISA and RDT have been completed. Some areas, particularly those that may be nearing interruption of transmission or those that the programme feels might be having problems, could be prioritized for early Phase 1 Ov-16 assessments. Country oversight committees would be ideal forums in which to prioritize these assessments. In areas where problems are not suspected or where LF programmes are planning transmission assessments in the near future, assessments could potentially be integrated with routine LF M&E or LF transmission assessments.

Monitoring and evaluation of programme impact is a vital component of the elimination effort. In an elimination programme, the need to strengthen programmes in areas that are not progressing as quickly as expected is urgent, because delays in addressing problem areas will delay interruption of transmission, unnecessarily prolonging the programme and delaying neighbouring areas or countries from completing PTS or PES. Although serological monitoring in children would be most consistent with the criteria for stopping MDA, monitoring prevalence in the entire population (e.g. including adults) could allow programmes to establish a community-level baseline that could potentially allow serology to be used as a component of PTS or PES, which may be more easily integrated into existing health systems than the current recommendations for vector monitoring. Monitoring should focus on those villages with the highest risk (e.g. first-line villages), although programmes may want to monitor additional villages, particularly if there are concerns about programmatic performance in certain areas.

Entomological evaluations with O-150 poolscreen PCR^23,24^ of blackflies in all TZs are recommended as part of M&E in Phase 1, but may not be immediately practical, particularly given the current laboratory capacity to support these evaluations. Although the goal would be to evaluate the annual transmission potential regularly as a marker of impact, the first steps could be to identify or confirm all the main productive breeding sites in the TZ, to identify the vectors at each site and to confirm the timing of the peak transmission season so that when the capacity to perform vector analyses regularly is present, these critical details will have already been determined. Once a country is able to implement PCR monitoring of vectors, the goal of monitoring could be to establish the trend of transmission in the flies, which should provide useful information to programmes.

One important M&E tool that was not described in the 2016 guidelines was the coverage survey. Often the first response to a programme identified as not progressing appropriately is the implementation of a coverage survey to determine if the reported coverage is equivalent to the actual therapeutic coverage (i.e. the treatment actually ingested). Good coverage is required for programme success, and higher coverage accelerates progress towards interruption of transmission.^14^ Fortunately, WHO in collaboration with partners has developed several draft coverage tools to assist programmes (www.ntdsupport.org/resources/supervisors-coverage-tool; www.ntdsupport.org/resources/coverage-survey-builder-coverage-evaluations). Programmes that cannot immediately implement other components of the M&E framework should consider coverage surveys as a way of identifying underperforming areas in the absence of other data.

## Optimizing elimination strategies

The data collected as part of new elimination mapping or M&E should be collected for the specific purposes of determining whether to initiate treatment or evaluating the appropriateness of the current treatment strategy. Models, such as ONCHOSIM or EPIONCHO, have the potential to provide information on project performance, but neither is designed to use current M&E data to project time to interruption of transmission for a TZ.^25^ Development of model algorithms that use changes in key indicators (e.g. Ov-16 seroprevalence in children or annual transmission potential) to project time to interruption of transmission could help programmes determine if a change in strategy is needed. In the absence of such algorithms, programmes will need to decide how they will determine if a programme is on track for elimination. There are five priority situations that need review to determine whether complementary treatment strategies are needed:

- Currently untreated endemic areas.
- Areas with high pre-control endemicity which models predict could not achieve elimination with once-yearly ivermectin MDA alone (e.g. community microfilarial load >70 microfilaria/snip^26^ or annual biting rates >10 000^27,28^).
- Areas not expected to achieve elimination by 2025 because of delayed start of MDA.
- Areas where demonstrated coverage over the last 3 years is insufficient to result in elimination by 2025.
- Areas not expected to achieve elimination by 2025 based on published models of onchocerciasis.

In any programme where there is an increase in prevalence indicators or a failure of those indicators to decline according to expectations, there is a need for careful review. Prior to adjusting a treatment strategy, the current strategy should be optimized. This would require performance of a coverage survey, evaluation of the operations supply chain at all levels and consideration of the optimal timing of MDA (e.g. at the beginning or peak of the transmission season).^29^ If the poor performance is shown not to be due to poor MDA coverage or timing of MDA, and other explanations cannot be found (e.g. recent immigration of infected populations), complementary treatment strategies could be considered.

Currently available complementary treatment strategy options include:

1. vector control; 2. increasing the frequency of ivermectin distribution, and 3. use of doxycycline.

- Vector control—reduction in the number of biting flies in the absence of vector elimination—when used in combination with ivermectin MDA will accelerate progress to elimination^26,27,30^ and may help overcome difficulties caused by systematic non-compliance. If this strategy were to be used in *Loa loa* co-endemic areas, it would also benefit those individuals who cannot receive ivermectin because of safety concerns. Novel methods of vector control, such as clearing rivers of the trailing vegetation required for successful vector reproduction, are in development and may result in vector control without the need for larvaciding (Dr Thomas Unnasch, personal communication).
- Increasing the frequency of ivermectin MDA to two or four times a year accelerates progress towards elimination by 40% or more,^31,32^ while increasing annual costs by around 60%.^33^ Depending on when a programme switches to this strategy, it may decrease costs or result in a marginal overall increase in cost.
- Doxycycline kills or sterilizes adult female worms.^34^ Used in combination with ivermectin, it could have potent impact on transmission by eliminating the source of new microfilariae, while ivermectin supresses transmission of microfilariae to blackflies. Unfortunately, doxycycline mass treatment would be expensive in the absence of a donation programme, would require good compliance with a weeks-long regimen and would require exclusion of pregnant women and children aged 8 years old and younger. Therefore, its use may be limited to areas in which the need to interrupt the transmission rapidly justified the expense, such as areas prone to instability. Additional research is ongoing either to shorten the doxycycline course, so that it could be given more easily in an MDA, or to find an alternative medication for treatment of adult female worms.

## Stopping MDA

The goal of an onchocerciasis elimination programme is to interrupt transmission by reducing the population of reproductively-capable worms, such that after interventions stop the population cannot recover. The evaluation required before stopping MDA before entering Phase 2 requires an assessment of recent transmission based on determining the seroprevalence of Ov-16 antibody in at-risk children younger than 10 years old and the prevalence of infection in blackflies as measured by O-150 poolscreen PCR.^13^ Children are evaluated in the seroprevalence surveys because a positive antibody test is thought to represent recent transmission. The current protocols for blackflies identify the prevalence of blackflies that are infectious to humans. This required human and blackfly testing is expensive and can be time-consuming, and many country programmes do not have easy access to laboratories that could perform Ov-16 ELISA and O-150 poolscreen PCR. Ideally, the presence of reproductively capable worms in humans could be directly measured. Unfortunately, no such test is currently available. Instead programmes measure other indicators of transmission that are correlated to the presence of adult female worms. Models and expert opinion have been used to set thresholds for these indicators, and this has been backed up by experiences in a few areas.^5,9,10,35–37^ Programmes will need to develop a systematic way of prioritizing which areas should undergo in-depth stop-MDA transmission assessments. One approach currently being studied is to perform a pre-stop-MDA survey (pre-SMS) that involves Ov-16 testing in a limited number of children in a few high risk villages. Programmes may want to consider using Ov-16 RDT for pre-SMS instead of ELISA in order to obtain more rapid results. Areas that pass the pre-SMS would then undergo the in-depth stop-MDA survey. Areas that fail the pre-SMS avoid the cost of the more in-depth assessment, while acquiring valuable M&E data. Although the threshold for passing the pre-SMS has not yet been determined, if a common strategy is used by multiple programmes the prevalences measured by programmes that passed both the pre-SMS and the in-depth stop-MDA transmission assessment could be used to define the pre-SMS threshold. Areas that were evaluated using OCP or APOC methodologies (e.g. in-depth skin snip surveys or entomological studies) may already have enough data to proceed with the stop-MDA transmission assessment without the need for a pre-SMS.

Unlike such a pre-SMS, the stop-MDA survey requires both epidemiological and entomological evaluations, and large sample sizes. In 2001, WHO established criteria for elimination that were operationalized in the Americas and then modified with the 2016 guidelines. The 2016 guidelines recommend that programmes demonstrate absence of transmission in humans using Ov-16 serology in at-risk children younger than 10 years old. The upper bound of the 95% CI of the seroprevalence must be less than 0.1%. The entomological indicator of transmission is the prevalence of O-150 PCR poolscreen positivity, although annual transmission potential can be used in certain circumstances. The upper bound of the 95% CI must be less than 0.1% in parous flies or 0.05% in all flies. The sampling methodology required for both epidemiological and entomological assessments is not fully described, though for the epidemiological evaluation a strategy of selecting local lower administrative units to create a representative random sample throughout the TZ is specified.^13^ Prior to the release of the 2016 guidelines, programmes used a variety of sampling strategies.^36–42^ Evaluations have been school-based^36,37,38^ and community-based.^40^ Communities have been selected randomly^37,42^ or through a two-stage approach in which a proportion of villages was selected from first-line villages and the rest was selected from among the other villages in the TZ.^41^ Within the villages or schools, children were selected randomly or conveniently. In some cases, children 10 years old and older have been included.^36^ The entomological assessments have varied as well, although flies were generally captured in breeding sites associated with first-line villages throughout the transmission season. In areas where the *Simulium damnosum* complex is not the principal vector, methods have had to be created that are in line with the specifics of the vector (e.g. monitoring of freshwater crabs that have a phoretic association with *S. neavei*). These varying evaluation designs merit further discussion as each has its own strengths and weaknesses. Operational research may be able to answer some questions about preferred methodologies and should be pursued. However, it should be expected that programmes will need to individualize their approach based on the epidemiological and entomological characteristics of the TZ. As defining the true extent of the TZ is difficult and as there are legitimate concerns about including hypo-endemic areas in evaluations of meso- and hyper-endemic areas, a two-staged sampling approach is likely to be required for the epidemiological evaluation. Part of the sample would derive from the first-line villages, which would historically have had the highest prevalence of disease, and the other part of the sample would derive from the rest of the TZ, which are the areas that programmes probably have the least information about. When determining where to sample blackflies, a central question is how to ensure that the selection of breeding sites is a good representation of transmission in the TZ. Ideally, programmes should take into account fly distribution throughout the TZ, biting rates at each site, predominant vector and geographic considerations that might require inclusion of a specific site.

## Cross-cutting issues

There are several important cross-cutting issues that onchocerciasis elimination programmes will need to address as part of their transition from programmes of control to programmes of elimination.

Particularly vital for programmes is effective data management. The data collected need to be readily available, regularly analysed and routinely used in making programmatic decisions. They also need to be stored in such a way that staff turnover does not prevent the programme from accessing the data, and they need to be easily available for inclusion in the dossier that programmes will need to submit as part of the process of WHO verification of the elimination of human onchocerciasis. The database should include not only baseline, surveillance and evaluation data, but also documentation of key programmatic decisions, including those operational decisions made with the advice of the national expert committee. Although there is ongoing discussion about the optimal data system, two potential systems include the District Health Information System 2 (DHIS2; www.dhis2.org) and the country integrated neglected tropical diseases database (CIND; www.who.int/neglected_diseases/data/ntddatabase/en).

Elimination targets also require programmes to pay close attention to the context of their work. Many areas are co-endemic with lymphatic filariasis, and integration of activities should occur whenever feasible (e.g. MDA, routine surveillance of both diseases simultaneously or of one disease during in-depth evaluation of the other). When integration is not possible, close coordination should occur. It is important to note that the need for continued treatment of lymphatic filariasis in an area that is ready to stop treatment for onchocerciasis and vice versa will affect the process. Although an in-depth evaluation to stop MDA for onchocerciasis can be performed in the setting of continued ivermectin and albendazole treatment, the process for post-treatment surveillance for onchocerciasis cannot begin until treatment for lymphatic filariasis has stopped.

Cross-border issues provide unique challenges to national programmes. Borders may be administrative borders within a country or international borders. In either case, programmes need to think about how to coordinate activities across these borders, as both people and blackflies cross them on a regular basis. Issues related to international borders are particularly complex, so programmes should not wait until they are ready to stop treatment to begin to develop the relationships needed to work with their colleagues in neighbouring countries. Data sharing will be important, and in some cases joint activities may be necessary (e.g. coordinated MDA and surveillance);^43^ this may require the creation of special intervention zones.

Domestic political will, accompanied by sufficient funding, will be needed to ensure that countries can maintain the focus on programme activities required to achieve the goal of elimination, particularly when the number of cases becomes low. Communities lose interest in a disease when they no longer perceive it as a threat, the cost per treatment rises and surveillance costs remain high. Obtaining political support early is important, and the formation of expert onchocerciasis oversight committees has the potential to help with this as they require programmes frequently to re-evaluate the impact of their activities. Demonstrating that the programme is making progress towards its elimination objectives and that it is closely monitoring its progress may help ensure that resources remain available.^44^ Additionally, publically celebrating programme success (e.g. through press releases or ceremonies) will draw regular attention to the impact of the programme.

## Conclusions

Transitioning the programme goal from onchocerciasis control to onchocerciasis elimination requires a shift in programme strategies that requires careful attention. Although the independent national programme oversight committees called for in WHO’s 2016 guidelines have an essential role to play in adapting global guidelines to local conditions, it is especially important that the challenges that these committees face and the approaches they adopt to meet the challenges be captured at the global level for dissemination to all endemic countries. In addition, since there are still uncertainties about how best to address programmatic issues—many occasioned by the shift from control to elimination targets—clearly articulating these uncertainties at the global and national levels will be necessary to define the operational research agenda needed to continue building the evidence base required to create guidance to assist programmes in determining which methodologies are best suited to their situations.
